# The Evolution of the Modern Hospital

**Published:** 1915-06-19

**Authors:** B. Burford Rawlings


### THE EVOLUTION OF THE MODERN HOSPITAL.*

### VIII.—The Problem of the Unpaid Staff.

By B. BURFORD RAWLINGS.

Human energies must be prompted by very strong impulses if they remain capable of persistence in the face of uncompromising opposition. They weaken of sheer weariness, and members of a managing committee who have started with an earnest desire to give their best powers to the cause they have espoused, find little encouragement to pursue their search for efficiency which, when achieved, nobody commends, and, if made operative, brings them into collision at once with a powerful body always on guard and quick to resent interference. Not the less we may reasonably affirm that a managing board which for lack of courage, patience, perseverance, knowledge, or any other quality helping towards efficiency abdicates one of the most important of its functions, is not rendering true and faithful service to the interests it has undertaken to safeguard and promote.

"The committee lives in terror of us" was the sarcastic comment of a physician quite disposed to uphold his professional rights and liberties, but sensible of the misfortune suffered by an institution when the management has become powerless in its dealings with the staff. In one instance, at least, a lay board was strengthened at medical instance against a threatened medical domination, but while a minority of hospital physicians are alive to the value of virile and educated lay government, it is equally certain that they find it difficult to separate themselves from their colleagues who think differently. On the other hand, the extremists, who customarily control a situation, have no desire that governing bodies should become strong and efficient. What do they fear? Lay authority in hospitals, salutary though its exercise, must be always more conspicuous in its restrictions than in its powers, and to be effective it must have the countenance and assistance of medical advisories. As now constituted the medical committee of a hospital is merely the medical staff under another name. The assistance needed by boards is of a medical body independent of the active staff, and naturally this is what the staff, so long as it is honorary, will oppose. The additional expenditure involved in a payment of the staff would be heavy, but with the help of the funds in aid it should not be formidable enough to prove prohibitive. The hospital appointment in itself is a valuable consideration, and any stipend would be supplementary to material advantages indispensable to the physician or surgeon who aspires to distinction. Hospital physicians seldom regard with favour proposals for adding to the numbers of the working staff. Yet an appreciable increase is called for. In all likelihood not an important hospital in London is manned adequately for the work to be performed. Physicians have been heard to say that to go the round of a dozen serious cases in the wards is an exhausting task. If we divide the number of beds in a given hospital by the number of the visiting doctors who have charge of in-patients we arrive approximately at the total of cases whose care each member undertakes. During holiday months, and in emergencies, this total is very largely exceeded. The statistics of the out-patients' rooms afford still more convincing evidence of stress. Recent legislation has resulted in considerable relief being afforded the overworked physicians, but the number of out-patients, when apportioned among the members of the staff who undertake their treatment, remains excessive, and patients too often are seen and prescribed for by students possessed only of a bare qualification and no experience. Not long ago a case was commented upon in the newspapers where an operation had been begun with fatal results. It was admitted that the patient was not in a fit state to receive an anæsthetic, and that due examination would have made this plain, but a medical witness is reported to have said that "there is no time at this hospital for examination unless some special reason is shown." To suggest that an increase of the staff would lead to the introduction of inferior men is not warranted. Eminence in any walk of life often indicates no more than ability to utilise opportunities, and doubtless the profession at large would be well pleased that the hospital door should be opened to a greater number of their body. The establishment of post-graduate classes was a wise step, but their usefulness would be increased if members were permitted a more intimate connection with hospital practice.

The chasm which separates lay and medical interests in some hospitals calls loudly for spanning. In the field of missionary enterprise highly satisfactory results have followed a combination of religious and medical training, and a medical missionary is regarded as the possessor of the best of all equipments for the exercise of his calling. What is possible and commendable in mission work ought to be attainable in hospitals. Some few important institutions are provided with a "medical superintendent," and it is generally understood that the presence of this officer helps materially towards comfort and economy. He holds office permanently. In age and position he has nothing in common with the transient house physician, or with the so-called "resident medical officer"; he is their senior and superior, and his professional standing permits him an authority in respect of them which quite rightly would not be tolerated in a layman. A development of the status, powers, and prerogatives of this office would go far towards providing a much-to-be-desired link between the two parties to the administration. If the officer we have in mind is to be valuable in the measure of his opportunities, independence must be secured to him, but he ought to be as acceptable to the staff as to the lay authorities, capable of impressing his

* Previous articles: February 20, March 6, 13, 20, 27; and April 3, 10, 17.

views upon both, and, while not concerned with the actual treatment of patients, he must be given a general jurisdiction in the wards. The management of a hospital thus modified would lose much of its difficulty. In the numerous and complex questions arising day by day, medical perhaps primarily, but intimately connected with general policy, the medical superintendent would make his influence felt and conciliate interests apparently antagonistic. The ideal hospital medical officer would need to forego some medical exclusiveness, to meet the layman in friendly spirit on common ground, and to allocate its rightful share of importance alike to philanthropy and science. Necessarily the personality of an officer of this description would be of supreme importance, and in its acknowledged dignity and emoluments the position must be made attractive enough to men of mark to induce them to hold office permanently.

The appointment of a medical superintendent or his equivalent in every hospital is no new suggestion. More than one expert has consistently advocated it. One answer of opponents—to quote words actually used—is that "the visiting staff would not for a moment brook interference," and no doubt that statement represents fairly the attitude of an honorary staff. It is, however, vital to the hope of amending the terms of medical service that whatever form the supreme government of the hospital may take it must be supreme also in the school, and that the staff should cease to be honorary. Then only may governing bodies look for a cordial co-operation of the staff. Then may we hope that voluntary effort will suffice to overcome the financial difficulties of which we hear so much, and some other shortcomings of which, perhaps, we are told too little.

Of this we may be sure, that if ever the voluntary system should be adjudged finally to have failed, the day of medical service as we know it will have departed, because no Government would venture to place before a democratic assembly a proposal to support or subsidise hospitals without taking the measures necessary to bring the members of staffs under control.

That some form of inquiry must precede a readjustment of lay and medical relations is unavoidable, and whether it be by Royal Commission or by any other means, one proviso is important to its efficacy—that all parties shall possess equality of representation, including the permanent officers, who live nearest to the centre of things, are witnesses of much which is hidden from persons in less responsible positions, and ought to be, of all men and women, the best informed upon the subject in its manifold bearings.

To return to the starting-point, and to urge that hospitals must be before all else philanthropic, is to reaffirm that they must not fail in power of attracting patients. If they are to remain dependent upon voluntary help they must be capable also of attracting benefactions. Neither patients nor benefactions would be forthcoming for organisations too demonstratively educational. Yet the work of the hospitals as teachers and demonstrators is of their essence. The only and inadequate alternative to their employment for teaching purposes would be the transfer of the schools to the State asylums and infirmaries, which do not depend for maintenance upon the good will of a small body of philanthropic givers. Were that done, the hospitals would buy some immunity, but they would be shorn of their greatness, their utility would cease to be world-wide, and the quality of their ministrations would deteriorate.

The difficulties of the task to be undertaken should enhance its inducements. To weld the professional and teaching side of hospital work with what is purely benevolent without detriment to either, and so to compose into a beneficent unity elements now not always in harmony, is a work worthy to exercise the powers of a master mind.

